# Palliative radiation therapy (RT) for prostate cancer patients with bone metastases at diagnosis: A hospital‐based analysis of patterns of care, RT fractionation scheme, and overall survival

**DOI:** 10.1002/cam4.1655

**Published:** 2018-08-17

**Authors:** Benjamin W. Fischer‐Valuck, Brian C. Baumann, Anthony Apicelli, Yuan James Rao, Michael Roach, Mackenzie Daly, Maria C. Dans, Patrick White, Jessika Contreras, Lauren Henke, Hiram Gay, Jeff M. Michalski, Christopher Abraham

**Affiliations:** ^1^ Department of Radiation Oncology Washington University School of Medicine St. Louis MO USA; ^2^ Division of Hospice & Palliative Medicine Department of Hospital Medicine Washington University School of Medicine St. Louis MO USA

**Keywords:** metastatic prostate cancer, National Cancer Database, palliative radiation

## Abstract

Prostate cancer (PCa) is one of the most common malignancies associated with bone metastases, and palliative radiation therapy (RT) is an effective treatment option. A total of 2641 patients were identified with PCa and bone metastases at diagnosis from 2010 to 2014 in the NCDB. Fractionation scheme was designated as short course ([SC‐RT]: 8 Gy in 1 fraction and 20 Gy in 5 fractions) vs long course ([LC‐RT]: 30 Gy in 10 fractions and 37.5 Gy in 15 fractions). Patient characteristics were correlated with fractionation scheme using logistic regression. Overall survival was analyzed using the Kaplan‐Meier method, log‐rank test, Cox proportional hazards models, and propensity score‐matched analyses. A total of 2255 (85.4%) patients were included in the LC‐RT group and 386 (14.6%) patients in the SC‐RT group. SC‐RT was more common in patients over 75 years age (odds ratio [OR]: 1.70, 95% confidence interval [CI] 1.32‐2.20), treatment at an academic center (OR: 1.76, 1.20‐2.57), living greater than 15 miles distance to treatment facility (OR: 1.38, 1.05‐1.83), treatment to the rib (OR: 2.99, 1.36‐6.60), and in 2014 (OR: 1.73, 1.19‐2.51). RT to the spine was more commonly long course (*P* < .0001). In the propensity‐matched cohort, LC‐RT was associated with improved OS (*P* < .0001), but no OS difference was observed between 37.5 Gy and either 8 Gy in one fraction or 20 Gy in 5 fractions (*P* > .5). LC**‐**RT remains the most common treatment fractionation scheme for palliative bone metastases in PCa patients. Use of palliative SC‐RT is increasing, particularly in more recent years, for older patients, treatment at academic centers, and with increasing distance from a treatment center.

## 1 INTRODUCTION

Prostate cancer is among the most frequently diagnosed malignancies in men and is one of the most common malignancies associated with osseous metastases.^1^, ^2^, ^3^ Since 2007, the incidence of metastatic prostate cancer at diagnosis has risen significantly.^4^ Patients who present with bone metastases at diagnosis often have significant pain or other skeletal‐related comorbidities. Radiation therapy (RT) is a safe and highly effective means to alleviate symptoms from bone metastases. Many different dose fractionation schedules have been reported, with 30 Gy in 10 fractions the most common schedule in the United States.^2^ Multiple prospective randomized trials and meta‐analysis have analyzed pain‐related outcomes with multifraction radiation therapy compared to single or shorter fraction treatments and have found similar pain control.^5^, ^6^, ^7^, ^8^, ^9^ Given these findings, and the potential to decrease the financial burden on the healthcare system, many medical societies including the American Society for Radiation Oncology (ASTRO) have released practice guidelines advocating single fraction or shorter course radiotherapy.^10^ In this study, we analyzed prostate cancer patients with metastatic bone disease present at diagnosis from the National Cancer Database (NCDB) to investigate patterns of care and overall survival. Of particular interest were changes in the patterns of care following ASTRO's bone metastasis evidence‐based guidelines and recommendations.^10^, ^11^

## 2 MATERIALS AND METHODS

### 2.1 Data source and study population

The NCDB Participant User File (PUF) for prostate tumors was reviewed to identify all patients between 18 and 90 years of age with a diagnosis of prostate cancer with bone metastases present at diagnosis. The NCDB is a joint program of the American College of Surgeons and the American Cancer Society. Data from approximately 70% of patients diagnosed at Commission on Cancer‐accredited cancer centers are included with patient, tumor, and treatment characteristics. Data elements are collected and submitted to the NCDB from commission‐accredited oncology registries using standardized coding and data item definitions, including RT dose/technique, chemotherapy use/timing, and Charlson‐Deyo comorbidity score. The Participant User File contains de‐identified patient and center information and was exempt from Institutional Review Board review.

De‐identified data for patients diagnosed with prostate cancer (PUF code: Primary Site = C619) who were diagnosed between 2010 and 2014 were evaluated. All included patients had bone metastases at the time of diagnosis (PUF code: CS Mets at DX‐Bone = 1) and were coded in the database as analytical Stage 4 (M1b). Demographic and clinical data included age, race, year of diagnosis, Charlson‐Deyo comorbidity index (CCI), treatment location and facility type, income, distance to treatment facility, primary insurance status, tumor T‐stage, and when available Gleason Score and PSA. Radiation treatment schedules were classified as short course (SC‐RT: 8 Gy in 1 fraction and 20 Gy in 5 fractions) or long course (LC‐RT: 30 Gy in 10 fractions and 37.5 Gy in 15 fractions). Only patients receiving RT to the bone were included (spine, ribs, hip, pelvic bones, shoulder, and extremity). We excluded patients who received radiation to the “Spinal Cord” who may have had spinal cord compression. Data regarding overall survival (OS) were available from patients with diagnosis between 2010 and 2013.

### 2.2 Statistical analysis

Descriptive statistics were used to analyze patterns of fractionation scheme use. The chi‐square test and Fisher's exact test were used to evaluate contingency tables, as appropriate. Logistic regression was used to assess for predictors of SC‐RT compared to LC‐RT. Variables with *P*‐values <.1 on univariate testing were entered into a multivariable analysis. Propensity score analysis was performed to correct for baseline differences between short‐course and long‐course groups. A matching algorithm including the variables used in univariate analysis, as well as receipt of hormone therapy and chemotherapy, was used with a caliper of 0.2. Exact matching was performed on the “treatment site” variable. Overall survival was calculated from diagnosis until death, censoring at last follow‐up for patients who were alive. The Kaplan‐Meier method was used to estimate overall survival probabilities and multivariable Cox regression was performed on all patients using the same variables as above. Significance was considered at a value of *P* < .05. All levels of significance were two‐sided. SPSS Statistics v.24 (IBM Corporation; Armonk, New York) was used for all statistical analyses.

## 3 RESULTS

### 3.1 Demographics and patient characteristics

**Figure 1:**
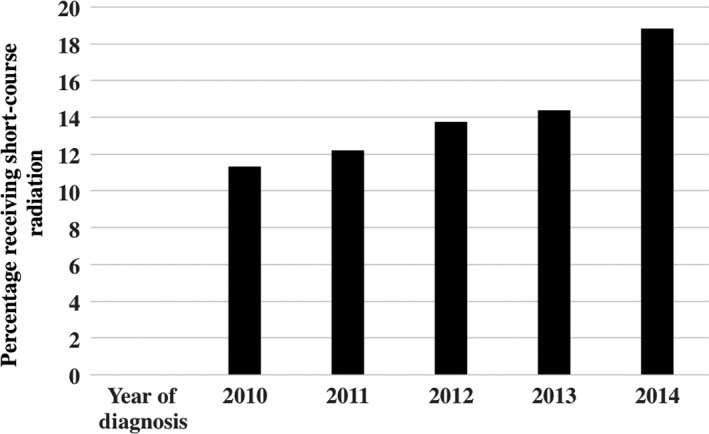
Distribution of short‐course radiation therapy (SC‐RT) by year of diagnosis (N = 2641 patients; 386 received SC‐RT)

A total of 2641 patients were included in the analysis. A total of 2255 (85.4%) patients were included in the LC‐RT group, and 386 (14.6%) patients were included in the SC‐RT group. One hundred and forty‐three (5.4%) received 8 Gy in 1 fraction, 243 (9.2%) patients received 20 Gy in 5 fractions, 1915 (72.5%) patients received 30 Gy in 10 fractions, and 340 (12.9%) patients received 37.5 Gy in 15 fractions. Median age of patients in the SC‐RT group was 73 years (range, 42‐90) vs 69 years (range, 39‐90) for the LC‐RT group. The frequency of SC‐RT increased from 11.3% in 2010 to 18.8% in 2014 (Figure 1). The most common site of bone metastases was spine (65.2%), hip (13.7%), pelvis (10.0%), and extremity (7.7%). 13.1% of patients living within 5 miles of their treatment facility received SC‐RT compared to 17.2% of patients living greater than 15 miles (*P* = .019). Complete details of patient characteristics are available in Table 1.

**Table 1:** Demographics and clinical characteristics

	Percentage of patients (#)		
	Short course	Long course	*P*‐value
Age			
<75 y	12.2% (207)	87.8% (1486)	**<.0001**
≥75 y	18.9% (179)	81.1% (769)	
Race			
White	14.1% (292)	85.9% (1774)	.246
Black	15.6% (70)	84.4% (380)	
Other	19.2% (24)	80.8% (101)	
Year of diagnosis			
2010	11.3% (44)	88.7% (345)	**.003**
2011	12.2% (55)	87.8% (396)	
2012	13.8% (70)	86.2% (439)	
2013	14.4% (85)	85.6% (506)	
2014	18.8% (132)	81.2% (569)	
Charlson‐Deyo comorbidity			
0	14.0% (284)	86.0% (1741)	.296
1	16.5% (70)	83.5% (354)	
>1	16.7% (32)	83.3% (160)	
Site of treatment			
Spine	11.8% (204)	88.2% (1518)	**<.0001**
Ribs	46.9% (15)	53.1% (17)	
Hip	17.4% (63)	82.6% (300)	
Pelvic bones	17.4% (46)	82.6% (219)	
Shoulder	21.8% (12)	78.2% (43)	
Extremity	22.5% (46)	77.5% (158)	
Facility type			
Academic/Research Program	19.7% (181)	80.3% (736)	**<.0001**
Community Caner Program	12.9% (37)	87.1% (250)	
Comprehensive Community Cancer Program	11.4% (128)	88.6% (994)	
Integrated Network Cancer Program	12.7% (40)	87.3% (275)	
Insurance status			
Medicaid	17.0% (38)	83.0% (186)	**.021**
Medicare	15.9% (234)	84.1% (1239)	
Not insured	12.0% (22)	88.0% (162)	
Other government	23.9% (11)	76.1% (35)	
Private	11.3% (76)	88.7% (594)	
Unknown	11.4% (5)	88.6% (39)	
Income			
<$38 000	14.4% (73)	85.6% (435)	.907
$38 000‐47 999	13.5% (84)	86.5% (536)	
$48 000‐62 999	15.3% (110)	84.7% (611)	
>$63 000	15.1% (117)	84.9% (659)	
Unknown	12.5% (2)	87.5% (14)	
Distance			
0‐5 miles	13.1% (117)	86.9% (775)	**.019**
>5‐10 miles	12.3% (79)	87.7% (561)	
>10‐15 miles	16.9% (56)	83.1% (276)	
>15 miles	17.2% (134)	82.8% (643)	

Bold values represent statistical significance between short‐course and long‐course radiation therapy (*P* < 0.05).

### 3.2 Univariable and multivariable logistic regression

On multivariable analysis, variables associated with increased likelihood of receiving SC‐RT included the following: increasing year of age (OR: 1.03, 95% confidence interval [CI] 1.01‐1.04; *P* < .0001), age group >70 years (OR: 1.56, (95% CI 1.25‐1.94); *P* < .0001), age group >75 years (OR: 1.70, (95% CI 1.32‐2.20); *P* < .0001), most recent year (2014) of diagnosis (OR: 1.73, (95% CI 1.19‐2.51); *P* = .004), year of diagnosis (2013‐2014) (OR: 1.50, (95% CI 1.11‐2.12); *P* = .01), treatment at an academic/research program (OR: 1.76, (95% CI 1.20‐2.57); *P* = .004), treatment to the rib (OR: 2.99, (95% CI 1.36‐6.60); *P* = .007), and living >15 miles from treatment facility (OR: 1.38, (95% CI 1.05‐1.83); *P* = .023). Regarding distance from treatment facility, with each increase in mile from the treatment center, there was an increased likelihood of receiving SC‐RT (OR: 1.01, (95% CI 1.00‐1.02); *P* = .040). Treatment to the spine was more commonly treated with LC‐RT (OR: 0.49, (95% CI 0.34‐0.71); *P* < .0001). CCI, insurance status, and income were not associated with receipt of either fractionation scheme. Complete details of both univariable and multivariable analysis are found in Table 2.

**Table 2:** Univariate and multivariate logistic regression for short‐course RT

	Univariate		Multivariate	
	Odds ratio	*P*‐value	Odds ratio	*P*‐value
Age				
<70 y	Reference Group		Reference Group	
≥70 y	**1.56 (1.25‐1.94)**	**<.0001**	**1.58 (1.30‐2.18)**	**<.0001**
<75 y	Reference Group		Reference Group	
≥75 y	**1.67 (1.34‐2.08)**	**<.0001**	**1.70 (1.32‐2.20)**	**<.0001**
Race				
White	Reference Group		—	
Black	1.12 (0.84‐1.50)	.436		
Other	1.44 (0.91‐2.29)	.119		
Year of diagnosis				
2010	Reference Group		Reference Group	
2011	1.09 (0.71‐1.66)	.692	1.06 (0.69‐1.64)	.786
2012	1.25 (0.84‐1.87)	.277	1.22 (0.81‐1.83)	.353
2013	1.32 (0.89‐1.94)	.165	1.30 (0.86‐1.94)	.192
2014	**1.82 (1.26‐2.62)**	**.001**	**1.73 (1.19‐2.51)**	**.004**
Charlson‐Deyo Comorbidity				
0	Reference Group		—	
1	1.21 (0.91‐1.61)	.186		
>1	1.23 (0.82‐1.83)	.318		
Site of treatment				
Extremity	Reference Group		Reference Group	
Spine	**0.46 (0.32‐0.66)**	**<.0001**	**0.49 (0.34‐0.71)**	**<.0001**
Ribs	**3.03 (1.41‐6.53)**	**.005**	**2.99 (1.36‐6.60)**	**.007**
Hip	0.72 (0.47‐1.11)	.133	0.79 (0.51‐1.22)	.290
Pelvic bones	0.72 (0.46‐1.14)	.161	0.77 (0.49‐1.24)	.289
Shoulder	0.96 (0.47‐1.97)	.908	1.15 (0.55‐2.40)	.719
Facility type				
Integrated Network Cancer Program	Reference Group		Reference Group	
Academic/Research Program	**1.69 (1.17‐2.45)**	**.005**	**1.76 (1.20‐2.57)**	**.004**
Community Caner Program	1.02 (0.63‐1.64)	.943	0.94 (0.58‐1.54)	.808
Comprehensive Community Cancer Program	0.89 (0.61‐1.29)	.529	0.88 (0.59‐1.29)	.505
Insurance status				
Not insured	Reference Group		Reference Group	
Medicaid	1.54 (0.85‐2.65)	.157	1.45 (0.81‐2.61)	.207
Medicare	1.39 (0.87‐2.22)	.166	1.08 (0.66‐1.78)	.757
Other government	**2.31 (1.03‐5.21)**	**.043**	1.78 (0.77‐4.12)	.180
Private	0.94 (0.57‐1.56)	.817	0.89 (0.53‐1.49)	.652
Unknown	0.94 (0.34‐2.65)	.913	0.95 (0.33‐2.72)	.926
Income				
<$38 000	Reference Group		—	
$38 000‐47 999	0.93 (0.67‐1.31)	.692		
$48 000‐62 999	1.07 (0.78‐1.48)	.667		
>$63 000	1.06 (0.77‐1.45)	.727		
Unknown	0.85 (0.19‐3.82)	.834		
Distance				
0‐5 miles	Reference Group		Reference Group	
>5‐10 miles	0.93 (0.69‐1.27)	.655	0.91 (0.66‐1.25)	.557
>10‐15 miles	1.34 (0.95‐1.90)	.095	1.31 (0.91‐1.87)	.143
>15 miles	**1.38 (1.05‐1.81)**	**.019**	**1.38 (1.05‐1.83)**	**.023**

Bold value represent statistical significance of the odds ratio between short‐course and long‐course radiation therapy (*P* < 0.05).

### 3.3 Outcomes

**Figure 2:**
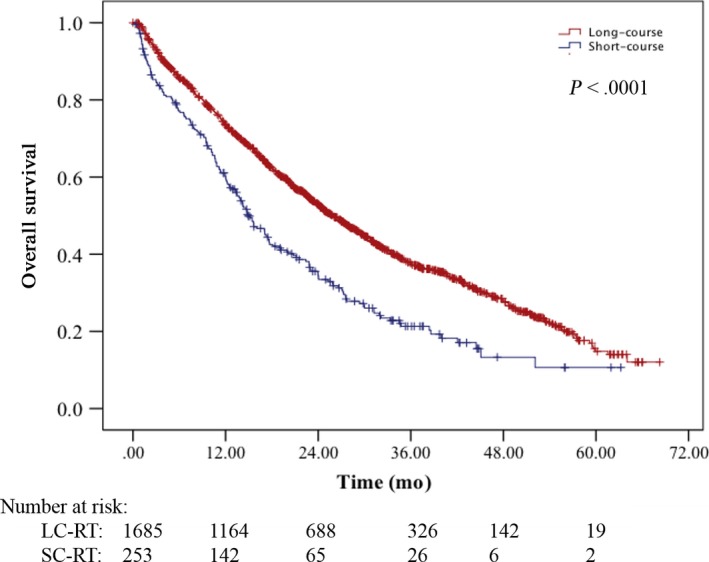
Kaplan‐Meier overall survival curve of patients treated with long‐course radiation therapy (LG‐RT) vs short‐course radiation therapy (SC‐RT). *P* < .0001

The median follow‐up was 19.2 months. The median OS of the entire cohort was 24.3 months (95% confidence interval [CI], 22.7‐25.8 months). Patients treated with SC‐RT had median OS of 14.9 months (95% CI, 12.7‐17.2) compared to 25.9 (95% CI, 24.1‐27.7) months for LC‐RT (*P* < .0001) (Figure 2). The median overall survival for patients treated with 8 Gy in a single fraction was 15.2 months (95% CI, 11.1‐19.3) vs 14.8 months (95% CI, 12.0‐17.6) for those treated with 20 Gy in 5 fractions (*P* = .832). There was also no significant difference in OS between those treated with 30 Gy in 10 fractions and 37.5 Gy in 15 fractions (*P* = .758).

### 3.4 Univariate and multivariable Cox analyses

On univariate analyses, OS was affected by length of radiation course (SC vs LC), receipt of hormone therapy, age, CCI, insurance status, distance to treatment facility, and clinical T‐stage (Table 3). On multivariable analysis, factors associated with worse OS included the following: short course of RT course (HR: 1.57 [95% CI, 1.34‐1.85]; *P* < .0001), no hormone therapy (HR: 1.36 [95% CI, 1.13‐1.63]; *P* = .001), age >75 years (HR: 1.70 [95% CI, 1.32‐2.20]; *P* < .0001), and CCI ≥ 1 (for >1, HR: 1.44 [95% CI, 1.17‐1.78]; *P* = .001).

**Table 3:** Univariate and multivariate Cox regression for overall survival

	Univariate		Multivariate	
	Hazard ratio	*P*‐value	Hazard ratio	*P*‐value
Fractionation scheme				
Long‐Course RT	Reference Group		Reference Group	
Short‐Course RT	**1.64 (1.40‐1.92)**	**<.0001**	**1.57 (1.34‐1.85)**	**<.0001**
Hormone therapy				
Yes	Reference Group		Reference Group	
No	**1.42 (1.19‐1.71)**	**<.0001**	**1.36 (1.13‐1.63)**	**.001**
Chemotherapy				
Yes	Reference Group		—	
No	0.94 (0.75‐1.18)	.617		
Clinical T‐Stage				
T1	Reference Group		Reference Group	
T2	1.04 (0.86‐1.25)	.676	1.00 (0.83‐1.20)	.970
T3	0.83 (0.51‐1.36)	.436	0.94 (0.73‐1.20)	.598
T4	**1.32 (1.05‐1.66)**	**.018**	1.26 (1.00‐1.58)	.054
TX	1.11 (0.78‐1.59)	.556	1.01 (0.70‐1.46)	.949
Unknown	**1.24 (1.06‐1.45)**	**.008**	1.17 (0.99‐1.37)	.061
Age				
<75 y	Reference Group		Reference Group	
≥75 y	**1.52 (1.41‐1.78)**	**<.0001**	**1.70 (1.32‐2.20)**	**<.0001**
Race				
White	Reference Group		—	
Black	0.92 (0.79‐1.07)	.292		
Other	0.78 (0.58‐1.05)	.099		
Year of diagnosis				
2010	Reference Group		—	
2011	0.90 (0.76‐1.05)	.177		
2012	1.0 (0.85‐1.17)	.969		
2013	0.93 (0.78‐1.10)	.400		
Charlson‐Deyo Comorbidity				
0	Reference Group		Reference Group	
1	**1.19 (1.02‐1.39)**	**.030**	**1.19 (1.02‐1.38)**	**.029**
>1	**1.59 (1.29‐1.95)**	**<.0001**	**1.44 (1.17‐1.78)**	**.001**
Site of treatment				
Extremity	Reference Group		—	
Spine	1.10 (0.88‐1.38)	.388		
Ribs	1.43 (0.83‐2.47)	.204		
Hip	0.88 (0.67‐1.15)	.340		
Pelvic bones	0.92 (0.70‐1.22)	.577		
Shoulder	1.29 (0.87‐1.92)	.210		
Facility type				
Integrated Network Cancer Program	Reference Group		—	
Academic/Research Program	0.86 (0.70‐1.04)	.124		
Community Caner Program	1.10 (0.87‐1.41)	.423		
Comprehensive Community Cancer Program	0.86 (0.70‐1.04)	.498		
Insurance status				
Not insured	Reference Group		Reference Group	
Medicaid	1.06 (0.78‐1.43)	.705	1.04 (0.77‐1.41)	.795
Medicare	**1.32 (1.05‐1.67)**	**.018**	1.09 (0.85‐1.34)	.499
Other government	1.06 (0.63‐1.78)	.836	1.02 (0.60‐1.72)	.946
Private	0.87 (0.68‐1.12)	.281	0.86 (0.67‐1.11)	.246
Unknown	0.98 (0.60‐1.62)	.939	0.88 (0.53‐1.46)	.167
Income				
<$38 000	Reference Group		—	
$38 000‐47 999	0.97 (0.82‐1.15)	.722		
$48 000‐62 999	0.94 (0.79‐1.11)	.445		
>$63 000	0.92 (0.78‐1.08)	.303		
Unknown	1.92 (0.99‐3.75)	.054		
Distance				
0‐5 miles	Reference Group		Reference Group	
>5‐10 miles	0.87 (0.75‐1.01)	.074	**0.85 (0.73‐0.99)**	**.042**
>10‐15 miles	**0.75 (0.62‐0.91)**	**.003**	**0.73 (0.60‐0.90)**	**.002**
>15 miles	0.92 (0.80‐1.06)	.251	0.92 (0.79‐1.06)	.230

Bold value represent statistical significance of the hazards ratio between short‐course and long‐course radiation therapy (*P* < 0.05).

### 3.5 Matched Cohort

**Figure 3:**
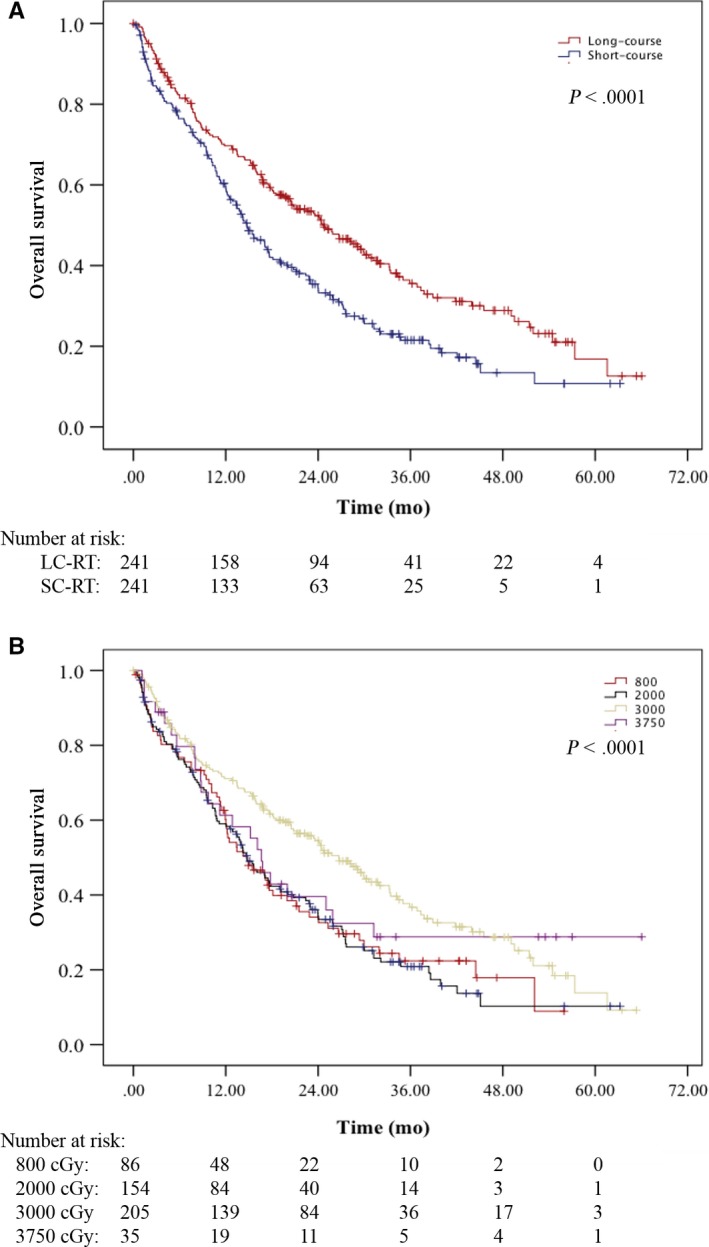
Propensity‐matched cohort. A, Kaplan‐Meier overall survival curve of patients treated with long‐course radiation therapy (LG‐RT) vs short‐course radiation therapy (SC‐RT). *P* < .0001. B, Kaplan‐Meier overall survival curve of patients treated with 8 Gy in 1 fraction (800 cGy), 20 Gy in 5 fractions (2000 cGy), 30 Gy in 10 fractions (3000 cGy), and 37.5 Gy in 15 fractions (3750 cGy). 8 Gy vs 20 Gy, *P* = .776; 8 Gy vs 37.5 Gy, *P* = .409; 20 Gy vs 37.5 Gy, *P* = .278; 30 Gy vs 8 Gy and 20 Gy, *P* < .0001; 30 Gy vs 37.5 Gy, *P* = .513

Propensity score matching between the SC‐RT and LC‐RT groups was performed to address confounding patient, tumor, and demographic bias between the groups. A propensity score match resulted in successful match of 242 pairs of patients between SC and LC‐RT (484 total patients). There were no significant imbalances in matched variables in the resulting cohort, and propensity scores were well matched (all standardized mean differences < 0.2). In the matched cohort, median OS for LC‐RT patients was 24.7 months (95% CI, 19.2‐30.3) vs. 14.8 months (95% CI, 12.4‐17.1) for patients treated with SC‐RT (*P* < .0001, Figure 3A). In analysis of the individual RT groups, there was no statistically significant difference in OS between 37.5 Gy in 15 fractions, 20 Gy in 5 fractions, and 8 Gy in 1 fraction (8 Gy vs 20 Gy, *P* = .776; 8 Gy vs 37.5 Gy, *P* = .409; 20 Gy vs 37.5 Gy, *P* = .278) (Figure 3B). The longest median OS was seen in those patients receiving 30 Gy in 10 fractions (30 Gy vs 8 Gy and 20 Gy, *P* < .0001; 30 Gy vs 37.5 Gy, *P* = .513). On multivariable analysis factors associated with worse OS included the following: short course of RT course (HR: 1.45 [95% CI, 1.22‐1.67]; *P* < .0001), no hormone therapy (HR: 1.38 [95% CI, 1.15‐1.69]; *P* = .001), age >75 years (HR: 1.75 [95% CI, 1.42‐2.29]; *P* < .0001), and CCI ≥ 1 (for >1, HR: 1.54 [95% CI, 1.31‐1.88]; *P* < .0001).

## 4 DISCUSSION

Prostate cancer is among the most common malignancies to metastasize to bone, and of those patients dying from prostate cancer, the rate of skeletal involvement ranges between 85 and 100% within 10 years of a metastatic diagnosis despite hormone therapy.^12^, ^13^, ^14^, ^15^ Bone metastases from prostate cancer are commonly associated with significant morbidity including pathological fracture, spinal cord compression, pain, and other skeletal‐related events. The current standard of care for palliation of pain and prevention of further morbidity caused by bone metastases is external beam radiation therapy. Multiple radiation fractionation schemes are used in the palliative setting with longer course (ie, >10 fractions) being more common in the United States.^2^ This is despite equivalent pain control and response rates with single fraction or shorter course treatments in several prior randomized studies and a large meta‐analysis.^5^, ^6^, ^7^, ^16^, ^17^, ^18^ Similar to prior reports, in this large hospital‐based study of patients with bone metastases at diagnosis, we found that ≥10 treatments remain the dominant fractionation scheme in the United States from 2010 to 2014 (85.4%).

Numerous clinical and demographic factors influence decisions pertaining to the choice of fractionation scheme in the treatment of bone metastases. These factors include, but are not limited to, many of the variables investigated in this study. We observed that those patients most likely to SC‐RT were those that were older, treated at an academic/research facility, received radiation o the rib, and lived over 15 miles from the treatment center. Additionally, we found that patients diagnosed in the most recent year evaluated (2014) were more likely to receive SC‐RT on logistic regression.

Multiple previous studies have observed that older patients are more likely to receive short‐course palliative radiation therapy.^2^, ^19^ This is likely due to the increasing medical comorbidities and declining performance status associated with increased age. While we incorporated Charlson‐Deyo comorbidity in our analysis, we did not appreciate an association between comorbidity score and selection of fractionation scheme. This is likely due to inherent difficulties capturing true comorbidity in the NCDB and lack of documented performance status. It can also be hypothesized that elderly patients likely have more difficulty in transportation to treatment facilities and are therefore more likely to receive SC‐RT. Our analysis found that patients residing more than 15 miles from a treatment facility were more likely to receive SC‐RT which supports this hypothesis. The trend toward more frequent use of SC‐RT also increased as the distance to treatment center was increased as well.

Our study also found that patients treated at academic/research facilities were more likely to receive SC‐RT than at nonacademic facilities. This finding is in keeping with studies in other disease sites, particularly breast cancer, that have reported earlier adoption of short‐course radiation therapy at academic centers compared to community practice as randomized trial evidence has emerged supporting the move to shorter radiation courses.^15^, ^20^ There are multiple potential explanations for this observation. Physician training and a lack of comfort with short‐course palliative radiation therapy, particularly single fraction treatment, clearly plays an important role in limiting the adoption of short‐course palliative treatments. These factors may be somewhat mitigated at academic centers in which the development and adoption of new treatment strategies are emphasized. It is unclear to what extent differences in reimbursement between short‐course and longer course palliative radiation influence behavior, but the current reimbursement model introduces bias in favor of longer courses of radiation. Differential reimbursement has been documented to influence choices among palliative treatments, such as palliative chemotherapy regimens, and this relationship is likely also present in radiation therapy decision making.^19^, ^20^, ^21^ Interestingly, our findings did not show any association between treatment scheme and patient insurance status, with uninsured patients no more likely to receive SC‐RT.

Anatomic location of bone metastases was also associated with the receipt of SC‐RT. Patients with rib metastases were more likely to receive a SC regimen and those with spine metastases more likely to receive a longer course. While we attempted to exclude patients with documented spine cord compression, this finding likely represents the use of LC‐RT for more durable control in the setting of spine metastases to prevent cord compression. This is supported by studies that have shown that the 18%‐20% retreatment rate in single fraction radiotherapy which is approximately double that of 10 or more fractions.^2^ It has been reported, however, that 16 Gy in 2 fractions or 8 Gy in one fraction are also effective in the treatment of metastatic cord compression.^22^

Another finding from this study is the increasing use of SC‐RT in the most recent time period examined. Schreiber et al^2^ published a similar study using the NCDB with metastatic prostate patients diagnosed between 2004 and 2012. In their study, longer fractionations schemes (≥10) were used in over 91% of patients. They did find, however, that year of diagnosis 2009 or later was associated with an increased likelihood of receiving SC‐RT. It may be considered that further increased use observed in our study coincides with multiple medical associations recommending shorter treatment courses. In 2011, Lutz et al^10^ published “Palliative Radiotherapy for Bone Metastasis: An ASTRO Evidence‐Based Guideline”. Following publication, it was noted to be one of the most downloaded *International Journal of Radiation Oncology, Biology, and Physics* articles each year for 4 years following publication.^23^ It was our hypothesis that following the release of these guidelines, a slow but apparent increase in SC‐RT would be appreciated. In our analysis, the frequency of SC‐RT increased from 11.3% in 2010 to 18.8% in 2014. In subgroup analysis, the rate of single fraction palliative radiation therapy also increased over the same time period (3.8% over 4 years).

However, there is ambiguity in the recommendations provided by ASTRO.^24^ The guidelines suggest that there is equivalent pain relief following 30 Gy in 10 fractions, 20 Gy in 5 fractions, or a single 8 Gy fraction, but do not frankly recommend adoption of short‐course or single fraction RT despite randomized evidence showing equivalence of the different fractionation schemes. For example, both the Choosing Wisely Canada campaign and The American Academy of Hospice and Palliative Medicine Choosing Wisely campaign are direct stating: “Don't recommend more than a single fraction of palliative radiation for an uncomplicated painful bone metastasis.”^25^, ^26^ Perhaps with a more firm stance from ASTRO adoption of shorter course RT would be implemented in the United States, similar to the more prevalent use seen in other countries such as Canada and the UK.^19^

In this study, we found that overall survival was superior in the group of patients treated with LC‐ RT on multivariable analysis. This overall survival finding is consistent with prior database reports, and is likely due to factors influencing the selection of radiation therapy treatment scheme that cannot be measured in the NCDB. In a propensity‐matched cohort, we continued to find an improved overall survival benefit to LC‐RT; however, in subgroup analysis, there was no overall survival benefit to 37.5 Gy in 15 fractions compared to either short‐course fractionation scheme evaluated. This conflicts the idea that more aggressive local palliative radiation therapy is associated with improved overall survival, and supports that patients selected for 30 Gy in 10 fractions likely have selection bias that cannot be measured in the NCDB.

Lastly, the adoption of single fraction or short‐course RT has significant financial implications for the radiation and medical community. A Dutch randomized controlled trial of 1157 patients with painful bone metastases compared both pain responses and cost of radiotherapy. They found no difference in pain response, and that the estimated cost of radiation therapy, including retreatments and other nonmedical costs, was significantly lower for a single fraction schedule compared to multiple fraction schedule ($2438 vs $3311, difference = $873, 95% confidence interval on the difference = $449 to $1297; *P* < .001).^27^ The Radiation Oncology Group (RTOG) found a similar financial advantage with single fraction over longer treatment courses in evaluation of RTOG 9714.^28^ In a New Zealand study, patient costs for single fraction RT were NZ$1344 (95% uncertainty level $855 to $1846) lower than multifraction RT for palliative bone metastases in the prostate cancer setting.^29^ Additionally, a recent SEER analysis reported a difference of $3094 (95% CI: $2107‐4081) between single fraction and 10 or longer fractionation schemes.^30^ There are estimates that an absolute increase of 10% use of single fraction palliative radiation therapy for metastatic prostate cancer could generate over $70 million per year in health cost savings.^2^, ^31^, ^32^

There are several limitations to this study, many of which are inherent to the NCDB and a retrospective analysis. The lack of clinical details regarding the specifics of each bone metastases is a major limitation. For example, degree of pain, number of metastases, complicated vs uncomplicated lesions, and additional courses of radiotherapy are all lacking from the database and likely significantly contribute to the selection of fractionation scheme. Additionally, while the NCDB codes for Charlson‐Deyo comorbidity, there are significant limitations to this variable in the NCDB and true comorbidities are likely vastly underestimated. The information regarding time to palliative RT is poorly coded in the database, and it may be possible that the length of life from time of metastatic diagnosis and need for palliative treatment may impact fractionation scheme of RT and survival. Lastly, there are no data in the NCDB regarding effectiveness or toxicity of RT, and overall survival is the only survival outcome available.

## 5 CONCLUSION

In this large observational study of the NCDB, the most common palliative radiation fraction scheme for men with prostate cancer and metastatic bone disease at diagnosis is ≥10 fractions. Despite recommendations from numerous medical societies and randomized data showing equivalent pain control with shorter treatment courses, LC‐RT remains the dominant fractionation scheme in the United States. Increasing age, treatment at an academic/research center, treatment to the rib, increasing distance to treatment facility, and diagnosis in 2014 were associated with increased likelihood of receiving short‐course RT. Increasing use of shorter fractionation schemes would provide significant costs reductions in healthcare spending.

## CONFLICT OF INTEREST

Authors have no conflict of interests.
